# "That's what you do for people you love": A qualitative study of social support and recovery from a musculoskeletal injury

**DOI:** 10.1371/journal.pone.0196337

**Published:** 2018-04-25

**Authors:** Khic-Houy Prang, Sharon Newnam, Janneke Berecki-Gisolf

**Affiliations:** 1 Monash University Accident Research Centre, Monash University, Melbourne, Victoria, Australia; 2 Centre for Health Policy, Melbourne School of Population and Global Health, The University of Melbourne, Melbourne, Victoria, Australia; UCL, UNITED KINGDOM

## Abstract

**Background:**

Social support has been identified as a significant factor in facilitating better health outcomes following injury. However, research has primarily focused on the role of social support from the perspective of the person experiencing an injury. Limited research has examined the experiences of the family members and friends of a person with injury. This study aims to explore the perceptions and experiences of social support and recovery following a transport-related musculoskeletal injury (MSI) in a population of injured persons and their family members and friends.

**Methods:**

This study was conducted using a phenomenological qualitative research design. In-depth semi-structured interviews were conducted with ten persons with MSI, recruited via the Transport Accident Commission (TAC) in Victoria, Australia. Seven family members and friends were also interviewed. The data was analysed using constant comparative method and thematic analysis.

**Results:**

Several themes were identified including: (1) key sources and types of support received, (2) relationship development and (3) challenges of providing and receiving support. Participants with MSI reported stories about how the social network provided emotional and tangible support. Family members and friends confirmed the supportive acts provided to the participants with MSI. Positive iterative changes in relationships were reported by the participants with MSI. Participants with MSI, their family members and friends described several difficulties including loss of independence, feeling like a burden, and the impact of caring on health and well-being.

**Conclusions:**

The role of social support is complex given the multitude of people involved in the recovery process. The findings of this study suggest that persons with MSI may benefit from support groups and maintenance of existing support networks. Furthermore, family members and friends engaged in the recovery process may benefit from support in this role.

## Background

Musculoskeletal injuries (MSI) are a major public health problem worldwide, contributing to a large burden of disability [1, 2]. According to the World Health Organisation’s Global Burden of Disease study, the majority of admissions for various non-fatal injuries as a result of a road traffic accident were related to MSI, with almost 50% of these injuries being fractures [3]. Beyond immediate health consequences, MSI can result in reduced quality of life, poor mental health, persistent pain, work disability and high medical costs [4–9]. The effects of MSI also extend beyond the individual to family members, friends, co-workers, employers, communities and societies [10–12].

Given the burden of MSI, identifying factors that can influence better health outcomes in the recovery process is essential. Social support has been identified as one of the significant factors in the recovery of MSI [13]. Social support is defined as information leading individuals to believe they are cared for and loved, esteemed and valued and belong to a network of communication and mutual obligation [14]. There are different types of social support that serve different functions. Types of functional support include informational (e.g. information about resources or advice), tangible/instrumental (e.g. assistance with transportation, cooking or financial resources), appraisal (e.g. affirmation/information relevant to self-evaluation) and emotional support (e.g. empathising, listening and caring). These types of social support can be provided by both formal and informal sources. Formal support can include the services provided by medical practitioners, self-help groups, supervisors and co-workers while informal support can be provided by social networks and community, such as family, parents, spouses, other relatives, friends, and peer groups [15].

Research to date suggests that people benefit physically and emotionally from having social support [16, 17]. Several studies have reported positive associations between social support and successful recovery outcomes among persons with MSI. For example, Nijs et al. [18] showed that persons with whiplash injury who received emotional, appreciative and informative support reported better long-term functioning outcomes than those who did not receive support. Similarly, Buitenhuis et al. [19] found that persons with whiplash injury who sought social support had shorter duration of neck complaints than those who did not. More recently, Prang et al. [13] and Baltov et al. [20] showed that among persons with MSI, better social support at work from employers and work colleagues was positively associated with return to work. Furthermore, studies conducted by Coronas et al. [21] and Holeva et al. [22] reported that among a road traffic accident population, lack of social support and perceived negative support (e.g. well-intended support perceived as unhelpful by the recipient) were associated with the development of post-traumatic stress disorder.

Despite advances made by researchers in examining the impact of social support on recovery outcomes, many gaps in the literature still need to be addressed. Social support is a bi-directional interactive process between the provider and recipient. The majority of quantitative [13, 18–20] and qualitative studies [23–27] have primarily addressed recovery from the injured person’s perspective and have not accounted for the interdependencies and transactional relationships between the social network. As MSI indirectly impacts family members and friends, further research is warranted to assess the interactions and effects of all persons involved in these supportive transactions. Furthermore, the existing qualitative research focused primarily on severe injury such as traumatic injury [23, 25, 28], spinal cord injury (SCI) [26] and traumatic brain injury (TBI) [27]. Given the severity of these injuries and the level of support required, it is unclear whether the results are generalisable to the MSI population.

In this study, we explore the role of social support and its impact on recovery from injury from the perspective of both the injured person and their significant others (i.e. family and friends). The significance of this research is in better understanding the type of support experienced by persons with MSI and the impact of this support on their family members and friends by comparing and contrasting their experiences post-injury. This research has the potential to lead to recommendations for a multi-level psychosocial intervention to better the health outcomes for those involved in recovery from injury.

## Methods

### Ethical considerations

Ethical approval for this study was granted by Monash University Human Research Ethics Committee (CF14/2232–2014001193). Written informed consent was obtained from all participants prior to data collection. All participants are referred to by pseudonym.

### Design

We undertook a qualitative study incorporating a phenomenological approach. Phenomenology seeks to describe how individuals experience a specific phenomenon. This approach characterises individuals’ lived experiences of a phenomenon through gathering extensive narrative data from a small number of participants. The goal is to generate a deeper understanding and meaning of a particular phenomenon from the individual’s perspective [29].

### Recruitment

Recruitment of participants occurred via the Transport Accident Commission (TAC). The TAC is a Victorian government-owned organisation that provides no-fault compensation to all persons injured in land-based transport accidents involving a car, motorcycle, tram, bus or train. No-fault benefits include medical treatment, income replacement, rehabilitation and long-term support services.

The TAC conducts a Client Experience Survey (CES) to measure client perceptions of TAC service delivery and to identify process improvements. Almost 1000 participants participated in the CES in October 2014 and February 2015. Following the completion of the CES, clients are asked if they would be interested in being contacted about future research; participants for the current study were drawn from this group of clients. Potential participants were purposefully selected according to the following criteria: a) sustained a MSI (i.e. dislocations, fractures, soft tissues including whiplash, sprains/strains); b) aged 18 years and over; c) 6–12 months post-MSI (note two participants were interviewed at 13–14 months due to the scheduling of the interview) and; d) having the ability to complete an interview in English. Seventy-five participants met the inclusion criteria and of those, 48 consented to be contacted about future research. TAC contacted the participants to gain consent for their contact details to be disclosed to the researchers. Researchers then contacted the participants who consented for their contact details to be disclosed and interviews were scheduled.

Interviews were conducted over a period of approximately one hour. Upon completion of the interview, each participant was asked to identify a family member or a friend who they felt had provided support to them during their recovery, regardless of whether they were the primary support person or not, to participate in the study. Each participant received a $25 gift card as compensation for their participation.

### Data collection

The data were collected by the first author through semi-structured interviews. The first author is an academic researcher with a psychological and epidemiological background, and worked in the compensation health research and health services research fields for a number of years. The majority of participants were interviewed face-to-face in their own home (n = 7) or at a convenient public location to the participants and researcher (i.e. café) (n = 6). Four participants were interviewed over the telephone due to rural location (n = 2), after hours availability (n = 1) and the telephone being the preferred mode of interview (n = 1). Interview questions were made up of a range of open-ended questions which aimed to explore the impact of social support on the injured person and the communication that take places among groups of interacting individuals (i.e. injured person vs. family members or friends) (Table 1). These open-ended questions intended to facilitate further exploration of the specific experiences identified by the participants. All interviews were audio recorded.

**Table 1:** Interview questions.

Participants with MSI	Family members and friends
*1. Tell me about your injury and recovery*.	*1. Tell me about your experiences dealing with your [injured family members*, *significant other*, *friend]*.
*2. Can you think of someone who you had the most interaction with following your injury? Tell me about your relationship with that person*.	*2. Did your relationship with the [injured family members*, *significant other*, *friend] change over the course of supporting their recovery? If so*, *how?*
*3. Did you encounter any positive or negative experiences from that person? If so*, *in what ways? How did it affect your recovery?*	*3. Was the [injured family member*, *significant other*, *friend] responsive to your own needs?*
*4. Did your relationship with that person change during the period of your recovery? If so*, *how?*	*4. Who helped you the most in your role? If so*, *in what ways?*
	*5. What was the most difficult or challenging?*

### Data analysis

Interview recordings were transcribed verbatim in a Word document, and imported into NVivo 10 for initial coding and storage. Constant comparative method and thematic analysis were used for identifying commonalities and points of divergence in the narrative between the different groups, and reporting patterns (themes) within the data [30, 31]. Patterns were identified through a rigorous process of data familiarisation, data coding and theme development and revision. First, the analysis process was initiated through familiarisation with the data, which involved several readings of the interview transcripts. Following this process, a coding guide (pre-set codes) was developed by the first author (KP), based on both familiarisation with the data and the relevant literature in the area of social support. The first author (KP) then analysed two interview transcripts using the initial coding guide and additional codes (emergent codes) were developed during the review of the data. These codes were then refined through discussion between the researchers (KP, SN). Two authors (KP, SN) then applied the codes independently to two interview transcripts. There was full agreement on application of all codes. The remaining interview transcripts were then coded by the first author (KP) using the final coding guide. For theme development and revision, similar codes were clustered together and subsequently collapsed into emergent themes by the first author (KP) and reviewed by the second author (SN). Given the sample size and heterogeneity of the sample (i.e. demographics and injury characteristics of participants), data saturation was not reached. However, purposive sampling, two independent coders, data source triangulation (i.e. constant comparative method) and consensus amongst the researchers were used to ensure trustworthiness of the results.

## Results

### Participants

Ten participants with MSI (21%) were recruited in the study. Half of the participants were male (n = 5) and ranged in age from 39 to 71 years. The participants’ marital status, injury types, hospitalisation status (proxy of injury severity), time since injury, educational level and employment status are detailed in Table 2. Seven family and friends that were nominated by participants were further recruited in the study. Six were family members and one was a friend of the participant with a MSI. Family members included mother (n = 2), spouse (n = 3), and daughter (n = 1). Family and friend participants were aged between 19 and 70 years of age. Two male participants without spouses did not nominate a family member or friend to participate in the study. The nominated family member of one participant declined to take part in the study.

**Table 2:** Demographic characteristics of the participants.

Participants with MSI (pseudonym)	Sex	Age	Injury type	Hospitalised*	Time since injury (months)	Marital status	Highest completed education level	Employment	Family member/ Friend participants (pseudonym)	Relationship with participants with MSI	Sex	Age
GEORGE	Male	71	Soft Tissue	Yes	8	Separated/ divorced	High school	Retired				
FRED	Male	42	Soft Tissue	No	14	never married	High school	No	SOPHIE	Mother	Female	70
CHARLOTTE	Female	39	Fractures	Yes	9	never married	Diploma	Working	DIANA	Mother	Female	69
EDWARD	Male	65	Fractures	Yes	10	married	PhD	Working (part-time)	MARY	Wife	Female	65
WILLIAM	Male	44	Dislocations	Yes	13	never married	High school	Working	HARRY	Friend	Male	47
SARAH	Female	50	Soft Tissue	Yes	6	married	PhD	Working	ANDREW	Husband	Male	57
CAMILLA	Female	59	Soft Tissue	No	9	married	Post-grad	Retired	ZARA	Daughter	Female	19
CHARLES	Male	52	Fractures	No	13	never married	High school	Working				
ELIZABETH	Female	65	Soft Tissue	Yes	7	married	Bachelor	Retired	PHILLIP	Husband	Male	60
ANNA	Female	56	Soft Tissue	No	10	separated/ divorced	High school	Working				

*No refers to care received in hospital without hospitalisation (i.e. a stay in hospital overnight or longer)

The qualitative analysis explored the role of social support and its impact on recovery from injury from the perspective of both the person with MSI and their family and friends. The findings are organised into three sections, each section denoting a theme. The themes arising from analysis of the interviews were: (1) key sources and types of support received, (2) relationship development and (3) challenges of providing and receiving support. In each theme, the findings concerning participants with MSI are presented first, followed by the findings concerning family members and friends. The findings in each theme are then compared across the two groups. Table 3 presents a summary of the three key themes for the person with MSI and their family and friends.

**Table 3:** Summary of key themes.

Themes	Participants with MSI	Family members and friend participants
Sources and types of support	Received • Parents • Spouses • Friends • Community (e.g. neighbours) • Employers • Work colleagues • Healthcare practitioners	Received • Spouses • Friends • Healthcare practitioners
	Received from sources of support • Emotional • Tangible • Informational	Provided to the persons with MSI • Emotional • Tangible Received from sources of support • Emotional • Informational
Relationship development	• Quality of relationship • Changes in family roles	• Minimal changes in the quality of relationship and roles in the family
Challenges	• Loss of independence • Sense of burden • Lack of social support	• Impact on health and wellbeing • Inability to plan • Carer duties and responsibilities

### 1. Key sources and types of support

Participants with MSI reported stories about how family members, spouses, friends, work colleagues and healthcare practitioners supported them following the transport accident. Similarly, family members and friends recounted numerous emotional and tangible support acts they provided to the participants with MSI. Each group’s experiences are discussed, below.

#### Participants with MSI

**Tangible and emotional support received from family members and spouses**

Participants with MSI who did not have a spouse relied heavily on their parents, particularly their mothers to provide tangible support. Tangible support they received included accommodation following the transport accident (i.e. from a week to indefinitely), meals, laundry services, medication management and transportation to medical appointments. Some of the participants reflected on their experiences with their mothers. For example, Charlotte recounted feeling appreciative of the emotional support received from her mother:

“Mum has been there for with me. My dad too, my dad is great but mum you know has been very supportive of me. In the last five years, I’ve actually had two lots of diseases. So, she’s the primary sort of you know person in my life that’s been there throughout the whole thing. So yeah she’s held my hand the whole time.” (Charlotte, 39 years old, fracture, admitted)

Participants with MSI who were married sought emotional and tangible support from their spouses. Married participants with MSI felt fortunate for consistently having their spouses around which allowed them to unreservedly share their concerns regarding the recovery. They also acknowledged their spouses for taking them to medical appointments. Edward described the tangible supporting activities undertaken by his wife, Mary:

“Yeah so she [wife] was actually back there by the time they’d cleared the ambulance to take me to the hospital. She didn't come in the ambulance so she followed the ambulance, so that was quite good. So, that was very helpful to have someone there doing the TAC claim, to do all the paperwork involved with it. So, I didn't have to worry about that too much, so for me that was all set up pretty quickly. So, in two days the claim was put in, the hospital was paid you know it was arranged so it was all done. So, she was quite good. She was coming to see me in hospital. She was also my driver. I wasn’t driving for a period of a few weeks.” (Edward, 65 years old, fracture, admitted)

**Tangible and emotional support received from friends and the community**

In addition, participants with MSI recounted how friends and the community, including neighbours, provided emotional and tangible support. Friends and community members visited the participants with MSI at home, provided words of encouragement and meals. For Camilla, the community was an important source of friendship:

“[Name of suburb] is that sort of place, it is quite community based and people pop in or call and so there’s a genuine concern for people’s wellbeing, which is lovely. Cards and people popping in and when the accident first happened a neighbour might bring over a bowl of soup or something like that so that he [husband] or the kids didn’t have to make a meal. Yes, so that additional community support/friendship support is very important, very strong.” (Camilla, 59 years old, soft tissue, non-admitted)

**Tangible and emotional support received from the workplace**

A number of participants with MSI who were working at the time of the injury expressed gratitude towards their employers and work colleagues for the emotional and tangible support they received. Participants with MSI perceived their employers to be understanding of the injury by allowing sufficient time off work to recover and ensuring that they did not return to work until they were ready. In addition, participants with MSI experienced visits from work colleagues whilst in hospital and received numerous well-wishes via telephone calls. Sarah spoke of the valuable emotional and tangible support she received from her work colleagues:

“Oh my colleagues were lovely because well they visited me because it’s a short walk from our offices to the ward. So, they came in and visited me you know about four different colleagues. And the lady I share an office with, she actually came when I was discharged and she actually expedited my discharge, because my blood pressure was a bit low so she got my blood pressure. But no, no look they were good and look there was a lady who, because my research assistant was away I actually had appointments so one of the other research assistants stepped in and she saw about six of my research participants while I was in hospital so that was a real help. Because it’s awful when your diary is full and there’s nobody you know it’s got to be taken care of.” (Sarah, 50 years old, soft tissue, admitted)

**Informational support received from healthcare professionals**

Other types of support recounted in the interviews included informational support. Participants with MSI received helpful informational support from their healthcare practitioners, including general practitioners (GPs), physiotherapists and psychologists throughout the recovery process. This was particularly important for those who did not have a spouse and had minimal contact with family members. Generally, participants with MSI were satisfied with the treatment they received and commended the healthcare practitioners for their professionalism and guidance. For example, Charles and Sarah spoke highly of the attentive care they received from their doctor and physiotherapist, respectively:

“Yeah the doctor…I’ve got a really good doctor. If I suggest anything or if he suggests something, it’s usually very effective and every time I see him he’s very attentive. And he’s very quick to refer if he thinks that I need somebody else’s advice. Yeah so I was quite happy with the doctor.” (Charles, 52 years old, fracture, non-admitted)

“The physiotherapist was the greatest help because you know I must have seen him about ten times because I had a very stiff neck and initially with very limited movement. And he kind of mobilised my neck and gave me exercises to do. And so even now I do those exercises at my Pilates class.” (Sarah, 50 years old, soft tissue, admitted)

### Family members/friends

**Tangible and emotional support provided to participants with MSI**

Family members and friends recounted similar tangible and emotional support acts provided to the participants with MSI. Family members confirmed their acts of tangible support through assisting the injured person with transportation to medical appointments and medication management. For example, Phillip kept a medication notebook to ensure his wife, Elizabeth adhered to a medication regimen. Family members also provided much tangible assistance to the participants with MSI with household chores (e.g. preparing meals and laundry services) and administrative tasks (e.g. TAC paperwork and payments). In addition, the majority of family members and friends recounted their experience of providing a great amount of emotional support. Diana spoke of the emotional support that she gave to her daughter, Charlotte, which she believed was necessary for the recovery:

“And I think from then on there she [daughter] just had to be encouraged and looked after you know. Just I think with me I think it’s just a bit of gentleness you know. If you’re gentle to yourself and you just you know keep yourself and say if I feel cranky today that’s okay too because I’m in pain. And when people are in pain they’re not exactly nice. So, it’s okay to be cranky it’s okay to have pain, it’s okay to cry and all those things, that’s what I’ve always encouraged her and she did that and she worked at getting better. That’s all you can do because ultimately getting better is you know something they work through.” (Diana, 69 years old, mother)

**Seeking emotional and informational support from spouses, friends and healthcare professionals**

In order to cope with the responsibility of providing care for the participants with MSI, several family members and friends stated that they reached out for emotional and informational support from their spouses, friends and healthcare practitioners. Seeking additional help ensured that they were looking after themselves whilst also being able to care adequately for the participants with MSI. For example, Diana recognised the crucial role her husband played in promoting her general health and well-being throughout her life:

“And you can always count on him [husband] you know to be there for your tears and me to be there for his tears. Anything you tell him he’ll always back you up and look after you. He will always. And that’s what she hasn’t got, that’s why I tend to give her a lot of time because I probably think of my life and how I’ve always got someone always and she hasn’t. So, it’s a great you know thing of support when you know your husband is always going to be there for you. I’m very lucky he’d be my biggest support. And because of him that’s how you can keep going. Because you tell him everything and he knows, he knows what it’s like.” (Diana, 69 years old, mother)

### 2. Relationship development

The theme of relationship development captures stories about how the relationship evolved following the MSI. The data identified changes in relationships that had a deep impact on both participants with MSI and their family members and friends. For participants with MSI, the focus of discussion was on the improvement of some relationships but not others, as well as breakdown of certain relationships, and role changes within the family. In contrast, family members and friends reported minimal change in their relationship with the participants with MSI.

#### Participants with MSI

**Quality of relationship**

Participants with MSI who did not have a spouse mentioned ways in which the injury strengthened the relationships with their family members, notably their mothers. Specifically, they recognised the importance of maternal support and how the injury brought the family closer, as reflected in Fred’s comment:

“Yeah it’s been good. We’ve always been close, Mum and I. It [relationship] got stronger. She’s been there when I’ve, well, pick up the pieces as they say. When I’ve been down and out. Yeah, Mum’s Mum, I love her. I won’t tell her that of course.” (Fred, 42 years old, soft tissue, non-admitted)

Although participants with MSI who were married received additional support from their spouses in the acute care, they did not believe that the relationships with their spouses changed much over the course of the recovery. The support they received from their spouses following the injury was consistent with support received prior to the injury, citing a strong bond with their spouses due to many years of marriage, as illustrated by Edward:

“Married for 34 years now. It’s quite good. Really intensively supported in the first ten days or so when I was off on leave. That was really about the only real sort of change. I needed her [wife] to drive me more than usual. Once I was back at work, past the whole six-week period, I’d recovered and there was really probably no real change you know in the relationship.” (Edward, 65 years old, fracture, admitted)

Despite positive development in some relationships, several participants with MSI who did not have a significant other at the time of the interview recalled stories about a breakdown in their relationship with their significant others following the accident. Charlotte described the loss of relationship and lack of social support received from her significant other over the course of her recovery:

“But as just the weeks went by I just felt like he [significant other] just became less interested and sort of expected me to get over it faster. And I don't know what happened, it’s a big mystery about this relationship but he broke up with me but he never officially broke up with me. I was with him about six weeks all up. He just sort of vanished one day and unfriended me on Facebook and you know blocked me as well that’s how we broke up. I was pretty upset about it all yeah.” (Charlotte, 39 years old, fracture, admitted)

**Family roles**

A number of participants with MSI also reported role changes within the family following the MSI. Role changes were related to issues of dependence and social support. Camilla expressed profound changes in her ability to perform satisfactorily in her role as a mother. She relied greatly on her spouse to perform her duties:

“Well in terms of me not being able to lift heavy things to clean, to vacuum. Certainly, for the first two to three weeks I wasn’t driving a car, our children needed…they were dependent on being taken places which would normally be my responsibility, pickups at night time and things like that. So, there were roles…my normal functioning within the relationship had changed and so he [husband] had to step up and take over those responsibilities and to a certain degree the children did too but for him more so it was him.” (Camilla, 59 years old, soft tissue, non-admitted)

#### Family members/friends

**Quality of relationship**

In contrast to MSI participants, family members and friends reported minimal changes in their relationships with the participants with MSI following the transport accidents. Mothers of participants with MSI reported always having a strong and close relationship with their children, regardless of the injury. Similarly, spouses described minimal changes in their relationships following the transport accidents. They believed that years of marriage created an intimate bond and loving relationship with their spouses. Mary described the authentic and enduring bond she has developed over the years with her husband, Edward:

“The fact that you actually really do need that person, and it’s good to know the relationship is so strong that they’re there for you. So that’s, I think, is a good thing that happened in our marriage. To know that it’s there and to feel grateful for having that strong, solid relationship. That you’re there for them through thick and thin. Which is the way it is. I know he’s [husband] there for me. That’s the good thing about being married so long. You’ve had that long companionship and friendship, and we know each other. I’ve had, you know, bad things happen to me, and he’s definitely been there for me, you know, when he has to be.” (Mary, 65 years old, wife)

**Family roles**

In contrast to the experiences of MSI participants who felt that their familial role had changed following the MSI, family members and friends reported no role changes within the family and social network. They believed the injury was not severe enough that it necessitated role change. Furthermore, they understood it was their role and primary responsibility as a loving mother, spouse, and friend to continuously support the participants with MSI to recover. Diana, Mary and Harry acknowledged this ethical responsibility towards the participants with MSI:

“Because she’s [daughter] not married and she’s got really, apart from her family you know they’re the people that you know she does depend on so that’s what you’ve got to do. It’s my role as a mother. And I mean I’m…that’s just the way it is you know.” (Diana, 69 years old, mother)

“That’s what you do for people you love. You do, you step up into the mark when they need you, and you don’t expect anything back. It was an additional burden I suppose, but it is part of my job as a wife, and as his partner. That’s what you’re signed in for.” (Mary, 65 years old, wife)

“Well you just don't even think about it. It's just something you do as a friend. As a moral duty, you could say.” (Harry, 47 years old, friend)

### 3. Challenges

The theme of challenges captured stories regarding difficulties in receiving and providing support following the MSI. Different challenges were experienced by the participants with MSI, their family members and friends. For participants with MSI, challenges revolved around loss of independence, self-perception of being a burden and lack of social support. In contrast, family members and friends expressed concerns with their own health and well-being, future planning and frustration with caring.

#### Participants with MSI

**Loss of independence**

Independence was raised by the majority of participants. These individuals reported they were appreciative of the support from their spouses and family members but also felt uncomfortable with their dependence on them. Elizabeth expressed frustration with her spouse Phillip when he thought she was unable to perform basic tasks and the impact this had on her mentally:

“He [husband] wanted to put me in cotton wool, because he knew I was in pain …increased pain because of the accident. It got to the point where I would sleep in this chair, he'd sleep in the bed inside. I became like a robot.” (Elizabeth, 65 years old, soft tissue, admitted)

**Sense of burden**

Feeling a sense of burden to others was common among the majority of participants. These individuals expressed concern about the physical and emotional burden on their spouses and family members that their injury would cause. George indirectly expressed distress about being a burden to his daughters. He attempted to reduce his burden on his daughters by justifying their behaviours and lifestyles. He described:

“Well we’re talking, we’re talking. They’re busy girls [daughters], they’ve got their problems, they’ve got their work, they’ve got high positions. So, they are busy they’ve got their families, they’ve got their little ones. I would say we call each other from time to time yeah. My daughters they don't know much about my suffering. They know I’m suffering, they don't know much about it.” (George, 71 years old, soft tissue, admitted)

**Lack of social support**

Some participants with MSI such as George, Elizabeth and Charles recognised the negative effects of not receiving support (i.e. isolation) from relatives and friends following the injury. Geographic locations and life circumstances including friends who are raising young families prevented them from accessing and receiving adequate forms of support from their social network. They expressed a desire for emotional and tangible support from their social network such as having someone to talk to, help with mowing the lawn, and transportation to healthcare services when required. Charles described the minimal support received from his friends:

“I haven’t had very much support no. I think it’s just the day and age that we live in and my age, a lot of my friends have moved away from the area. And my remaining friends are very locked into their work so we don't communicate very often these days.” (Charles, 52 years old, fracture, non-admitted)

#### Family members/friends

**Impact on health and well-being**

The challenges experienced by family members were different to that of MSI participants. Family members raised concerns regarding their health and well-being. They revealed that caring for the participants with MSI was demanding at times and impacted their health especially if the family member was in paid employment. For example, Phillip felt he had to quit his job to become Elizabeth’s full-time carer. Diana and Phillip also developed anxiety and depression, respectively, which they thought may have been directly linked to caring.

“He’s [doctor] just given me something to take the edge off. I don't know whether it makes you worry less but it helps you cope with it. Not that you’re asleep all the time or anything like that, just to take the edge off my anxiety so that I can cope a bit better. You sort of think oh well this is what I’m going to do because if people depend on you and then you go to pieces.” (Diana, 69 years old, mother)

**Inability to plan**

Furthermore, family members expressed concerns with the endless medical appointments which disrupted their lives and made it very difficult to plan for the future. This was reflected in Sophie’s comment regarding her son’s medical appointments and the inability to go on a vacation:

“Well, we’re ready to go down there again but he’s [son] still got more important appointments at the moment. He’s got to get sort of out. They’ve [doctors] now diagnosed him with a disease. Once he gets over these next appointments, hopefully we’ll book a holiday but I’m running out of time. I don’t like going in the cold weather. It’s March and he’s still got appointments into April.” (Sophie, 70 years old, mother)

**Caring duties and responsibilities**

Finally, family members and friends felt helpless observing the participants with MSI in pain or unable to complete basic household chores (e.g. making the bed and vacuuming). They expressed a desire to help with the recovery of the participants with MSI but became frustrated when the participants with MSI did not want assistance, as illustrated in Phillip’s comment:

“An uphill battle. As you saw Elizabeth is very strong willed to the point of being detrimental to her situation in that she tries to take too much on like vacuuming, she’s always at the risk of falling. It’s one of the problems in being disabled, people don’t like to be disabled so they try to push themselves to do, some people, more than they should or can really do, that having dire consequences. I mean Elizabeth says I’m overprotective or I’m authoritarian but I can see from past actions what will happen if a certain thing is done. You can get very frustrated and the anger can bubble up. Now I just try to take a couple, four deep breaths and try and zone out.” (Phillip, 60 years old, husband)

## Discussion

### Main findings

This qualitative study explored the perceptions and experiences of social support among people with MSI following a transport accident, their family members and friends. The findings suggest that the role of social support is complex given the multitude of people involved in the recovery process. Furthermore, the results revealed similarities and contrasting viewpoints among participants with MSI, their family members and friends across three themes: (1) key sources and types of support received, (2) relationship development and (3) challenges of providing and receiving support.

The results of this study show that different members of the social network provided different types of support. Among participants with MSI, spouses and mothers undertook activities to manage the MSI recovery including physical care, meals, transportation to healthcare services and emotional support. This support was important and highly valued by the participants with MSI, particularly in the acute stage of recovery when they were unable to care for themselves. The types of support reported are consistent with past qualitative studies conducted among traumatic injury, TBI and SCI populations [26, 32–34].

In contrast, participants with MSI who had minimal contact with family members relied on healthcare providers for informational support regarding their MSI prognosis and treatment. Similarly, past research showed that people with illness who reported no spousal support were more likely to access formal and professional support services for daily care and illness management than those with a spouse [35]. The findings suggest that healthcare practitioners need to take into account the social circumstances of persons with MSI, especially when they have limited social support and are unable to care for themselves to ensure optimum recovery.

Relationship changes after the MSI were evident in some relationships but not others. Participants with MSI who relied heavily on support from their mothers developed stronger relationships with them following their MSI. This finding supports previous work conducted among people with traumatic injury, in which the injury resulted in a strong recognition of the importance of family post-injury [23]. The stronger bond is likely due to an acknowledgment, recognition and sense of gratitude for their mothers’ unwavering commitment and support in time of needs. In contrast, no changes in the relationship with their children were reported by the mothers. Their willingness to assume care for their children is derived from their feeling of love and familial ethical responsibility, as noted in previous traumatic injury and TBI studies [27, 32, 36].

In contrast to the parental and child relationship, no changes in the relationship dynamics were reported among participants with MSI who had a spouse and by their spouses. The quality of the relationship was considered relatively similar prior to the MSI. Our findings do not confirm previous research in the TBI and SCI populations whereby spousal relationships changes are evident [33, 37, 38]. This may be due to the severity of the injury. MSI were viewed by the participants and their spouses to be a relatively minor injury despite a number of participants requiring hospitalisation. Furthermore, marriage is considered a unique relationship because of its intensity, duration, and dependence on each other; in this study, the couples were married for a substantial period of time. They were also likely to have previously experienced a number of crisis (results not shown) and therefore may have learnt to adapt and manage their problems successfully by depending on each other.

Some participants with MSI had experienced some degree of relationship breakdown with their significant other following the transport accident. Although it was unclear whether the breakdown of the relationship was attributed to the injury, this could suggest that MSI can put severe stress on relationships. Past research has shown the rate of relationship breakdown to be relatively high following TBI and SCI [37, 38]. In support, evidence from the family members’ interviews revealed that one of the major challenges for them was the emotional burden of caring for the injured person and this had an impact on their health and well-being. To minimise relationship breakdown, we recommend that information regarding the injury recovery process including potential changes in responsibilities and role be provided to partners of persons with MSI by rehabilitation healthcare practitioners. In addition, couple counselling for severe cases would allow the partner to gain further insight and understanding of the impact an injury has on a person, and how to manage and overcome a potential life changing injury together.

A number of challenges and difficulties were identified by the participants with MSI including loss of independence and concerns about being a burden. Participants with MSI also raised issue with the limited amount of support provided by their social network. Injury deprived individuals of independent performance of self-care and household tasks. As identified in the interviews, spouse and family members recounted playing a central role in completing household tasks such as preparing meals and doing the laundry. Some participants with MSI return to the family home despite having lived away from home prior to the MSI. This represented a huge loss of independence and self-reliance, as described in previous traumatic injury and TBI studies [23, 39]. With a better understanding of the consequences of injury and possible role change after injury, healthcare practitioners can target the development of specific skills necessary for the continuation of valued roles.

Participants with MSI also shared common concerns of being a burden on their spouse and family members. Research in the traumatic injury, TBI and SCI populations supports the view expressed by participants with MSI [23, 26, 39]. However, there was evidence from the family members and friends’ interviews that they perceived their relationship with the injured person to improve over time, regardless that the injured person thought they were a burden. Burden on the family may prevent people from seeking and receiving support, thereby potentially hindering their recovery. Thus, further study is required to explore which strategies are most effective in decreasing persons with MSI’s worries and fears about being a burden on their families and friends.

Although the majority of the participants with MSI received support from their spouses, family members and friends, several participants with MSI reported not receiving sufficient support and feeling lonely. A lack of social support can result in social isolation. Long periods of loneliness can have a negative impact on physical and mental health [40]. This study proposed that the maintenance of current social support network (i.e. connecting/reconnecting with family and friends) and the development of new social support system (e.g. online support groups), particularly for those with limited support is essential to prevent loneliness and isolation [41, 42]. In addition, training for persons with MSI to understand the type of support that is required in a particular situation and identify the people within the social support network who are able to provide it will be imperative for recovery [42]. Training may include informative video and written information (e.g. social support guide).

Spouses and family members experienced a variety of problems including physical and mental ill health, and a lack of support. Caring was perceived as demanding, emotionally taxing and physically draining by some of the spouses and family members. Some carers suffered from both physical and mental health conditions which may have arisen as a direct result of providing care. Similarly, in past studies, caring for an injured family member has been associated with poor general health and mental well-being [33, 36]. However, not all family members experienced problems, and some were able to make adjustments, particularly when they perceived the MSI to be relatively minor and when familial roles did not alter. The findings suggest that to differing extents, some spouses and family members will require support to help them cope and prevent burnout. Future studies are needed to further explore the needs of the carer, the physical impact of caring and identify which carers are at higher risk of injury and poor health and in need of support and training.

### Strengths and limitations

Although this study provides greater insights into the role of social support following a transport accident from the perspective of the persons with MSI, their family members and friends, there are several limitations that should be considered when interpreting these study’s findings. The recruitment was based on participants with MSI who completed the CES and agreed to be contacted for future research, which may have resulted in an inclusive biased sample (i.e. selected for convenience). However, the inclusive biased sample (e.g. older, married and hospitalised) does not undermine the study’s findings as we recognised that the sample is not fully representative of the MSI population injured in road traffic accidents (e.g. younger and not hospitalised). We also recognised that individual experiences after MSI can vary considerably. Therefore, our small sample may not have captured the complete range of views about how social support affects recovery. Further research is required to assess whether the themes suggested by our sample are similar in a broader sample with various age groups and life course experiences (e.g. employed vs. retired, dependent children vs. no children). We did not have information on the severity of MSI, the psychological status of the persons with MSI, their family members and friends which may have impacted injury recovery and the type of support a person with MSI received. However, hospitalisation has been included as a proxy for injury severity. The transferability of the findings needs to be considered in this light. Furthermore, due to ethical constraints we were unable to directly approach family members and friends for interviews. Only the nominated family members and friends who were considered to be supportive during the recovery process were interviewed, which suggest that the participants with MSI may have had more supportive relationships. Interviewing family members and friends who were unsupportive would have provided insight into why certain people are unable to provide support (e.g. uncertainty about the most effective way to provide support, limited ability to provide support under stressful circumstances, pressure on relationship) and would potentially lead to identification of strategies for providing support. Using a focus group methodology with a larger sample may help overcome the recruitment limitations imposed from this study.

## Conclusions

In conclusion, this qualitative study provides a greater understanding of how social support is perceived and experienced by people with MSI, their family members and friends. Three themes were identified: (1) key sources and types of support received, (2) relationship development and (3) challenges of providing and receiving support. For both participants with MSI, their family members and friends, emotional and tangible supports were considered important for facilitating recovery from MSI. Changes in some relationships were evident among participants with MSI. However, these relationship changes were not perceived by the family members and friends. For participants with MSI, challenges included independence, feeling like a burden and lack of support, whereas for family members and friends, the difficulties associated with caregiving were health and well-being and future planning. The study suggests that the development of support groups and maintenance of support is particularly critical for those with limited or no support. Supporting family members and friends’ continued engagement in the recovery process is essential especially for carers whose health is compromised.

## Abbreviations

CES: client experience survey
GP: general practitioner
MSI: musculoskeletal injury
SCI: spinal cord injury
TAC: Transport Accident Commission
TBI: traumatic brain injury
